# Policy makers, the international community and the population in the prevention and treatment of diseases: case study on HIV/AIDS

**DOI:** 10.1186/s13561-016-0139-x

**Published:** 2017-01-25

**Authors:** Kjell Hausken, Mthuli Ncube

**Affiliations:** 1 Faculty of Social Sciences, University of Stavanger, 4036 Stavanger, Norway; 2 Blavatnik School of Government & Fellow, St Antony’s College, University of Oxford, Radcliffe Observatory Quarter, Oxford, OX2 6GG UK

**Keywords:** Disease, Policy, Game, Funding, Prevention, Treatment, Resource distribution, Free riding, Risky behavior, C72, D72, D74, I10, H51

## Abstract

A four-period game is developed between a policy maker, the international community, and the population. This research supplements, through implementing strategic interaction, earlier research analyzing "one player at a time". The first two players distribute funds between preventing and treating diseases. The population reacts by degree of risky behavior which may cause no disease, disease contraction, recovery, sickness/death. More funds to prevention implies less disease contraction but higher death rate given disease contraction. The cost effectiveness of treatment relative to prevention, country specific conditions, and how the international community converts funds compared with the policy maker in a country, are illustrated. We determine which factors impact funding, e.g. large probabilities of disease contraction, and death given contraction, and if the recovery utility and utility of remaining sick or dying are far below the no disease utility. We also delineate how the policy maker and international community may free ride on each other’s contributions. The model is tested against empirical data for 43 African countries. The results show consistency between the theoretical model and empirical estimates. The paper argues for the need to create commitment mechanisms to ensure that free riding by both countries and the international community is avoided.

## Background

The global burden of disease is substantial. The prevalence of diseases wax and wane, and vary across countries and through time regarding the need to distribute funds. This paper focuses on diseases where funds are allocated for prevention and treatment, after which nature chooses a population fraction that contracts the disease. Treatment causes some to recover while others remain sick or die.^1^ This papers tries to answer a few questions, as a way to get to some policy solutions: Why do some countries free ride on their responsibility to treat their citizens? Why do some donors prefer to focus on funding preventions when treatment is also a form of prevention? Which factors determine which population fraction contracts the disease? To what extent is the amount of funding committed consequential in changing the population fraction contracting the disease? Are the current mechanisms in place to make sure both policy makers in affected-countries and donors commit to funding both treatment and prevention of diseases?

In order to answer these questions and get to some solutions, the paper uses a game-theoretic approach involving policy makers, international donors, and people living with diseases. The behavior of the parties is driven by reactions to each other’s actions, which results in outcomes that the questions above imply. The paper goes to the heart of the need to create more binding commitment-mechanisms to counter free riding by affected countries and also donors, in the financing of disease prevention and treatment. The paper also fleshes out the behavioral incentives for those living with diseases and those who could contract it, pointing to the need for to meet their side of the bargain.

The paper assumes that policy makers and donors have accepted the *duty of rescue* and the people also have a *duty to respond* to rescue efforts, see Collier et al. [9]. Collier et al. [9] discuss the implications of the duty of the rescue principle on treatment and prevention choices, and the size of lifecycle financial liabilities for disease intervention and its fiscal implications. Other approaches, such as *accountability for reasonableness*, lend support to prioritization decisions by policy makers and donors in their interventions, see Daniels and Sabin [11]. Accountability for reasonableness seeks to establish a framework for prioritization and fair allocation of responsibilities in decision-making, unlike considerations of cost-effectiveness and comparative effectiveness research.

Historically in disease development, individuals moved first spreading the disease, through mechanisms such as infection, air transmission, food distribution, and lifestyle imitation. Policy-makers and others moved thereafter reacting to the disease. Finally individuals moved again, either increasing or decreasing their risky behavior. In this paper we do not focus on the history, but on what to do as we move into the future. To design a tractable model we thus omit the first step and start by considering what a policy maker should do here and now as we move into the future. A four-period game-theoretic model with complete information is conceptualized. First, a policy maker associated with the government in a country distributes funds between disease prevention and disease treatment. The fraction of funds distributed to disease prevention impacts whether individuals contract the disease. Second, observing the policy maker’s available funds and distribution, the international community provides an analogous distribution between prevention and disease treatment. The international community^2^ is considered to move second since in many countries, with adequate funds and distribution, intervention by the international community is not needed. In contrast, in other countries either lacking funds or inadequate distribution suggests the need for the international community to intervene.

From the model we are able to generate various properties which we discuss. Furthermore, from the outcomes of the model, the paper argues for the need to create commitment-mechanisms to ensure that free riding by both countries and donors is avoided. Although a cooperative game with binding agreements between the players would be desirable, such a game is hard to implement and sustain. Hence in this paper we develop the more fundamental non-cooperative game, with no binding agreements between the players, to illustrate the dilemmas. The paper also argues for commitment to funding both prevention and treatment, by policy makers and donors. Without these mechanisms the game will result in countries with limited resources only focusing on prevention, which is not desirable. The model also shows why more funding is needed, and how that can reduce the probabilities of disease contraction, and death from the disease.

The paper also addresses fiscal sustainability and debt sustainability issues. If, for example, donors free ride, how could a country meet its future unfunded debt from disease liabilities. The fiscal stability of any country, with or without resources is threatened.

The model is tested using HIV data for 43 African countries which are classified according to the model’s characteristics. We test the model using regression analysis. Policy implications, suggestions and predictions and presented.

Health policy decisions are usually analyzed "one player at a time", which has various disadvantages associated with sectorial analyses and non-holistic analyses which may not capture phenomena comprehensively. Sectorial analyses may be incorrect when relevant cause-effect relationships are shut out from consideration. In this paper we bring the relevant players together in a game-theoretic approach to account for their different interests in a holistic approach.

Examples of disease prevention are awareness campaigns so that people are knowledgeable and take precautions e.g. by taking vaccines against disease contraction, or using condoms to avoid HIV contraction. Examples of disease treatment are hospital beds and medicines to treat diseases, and ameliorate the adverse effects of diseases, given that disease contraction has occurred.

Resource allocations between prevention and treatment are sometimes seen as being in competition. Literature abounds on decision-making for resource allocation for disease treatment and prevention. For HIV see Paltiel and Stinnett [27], Marseille et al. [25], Gonsalves [14], Kumaranayake et al. [23], Canning [6], Alistar and Brandeau [1], Boily et al. [4], Bärnighausen et al. [5], Bertozzi et al. [3], and the HIV Modelling Consortium Treatment as Prevention Editorial Writing Group [17], among others. There is also quite some focus on cost-effectiveness analysis of prevention measures, see Walker [29], Hogan et al. [18], Goldie et al. [13], Cohen et al. [8], Creese et al. [10], Galárraga et al. [12], and Granich et al. [15]. See Izazola-Licea et al. [19] and Hecht et al. [16] regarding financing the response to HIV/AIDS, and Coates et al. [7] regarding behavioral strategies for reducing transmission of the disease.

Some of the literature has focused on the incentives for resource allocation by corporations to develop drugs for either prevention (vaccinations) and treatment. See Alistar and Brandeau [1] for decision making for HIV prevention and treatment. Private incentives for developing treatment drugs seem far stronger than those for developing vaccines for prevention, see Thomas [28], Kremer and Glennerster [20], Kremer and Snyder [21, 22], Kremer and Snyder [22], inter alia. Countries with high disease prevalence may be forced to allocate large budgets to disease treatment which may leave little left for disease prevention. This paper throws light on such resource allocations. See Mamani et al. [24] for a game theoretic model of international influenza vaccination coordination, and Moxnes and Hausken [26] for mathematical modeling of acute virus influenza A infections.

The rest of the paper is organized as follows. Section 2 presents the model. Section 3 analyzes the model. Section 4 compares the model with empirics. Section 5 concludes.

## Methods: The model

**Fig. 1:**
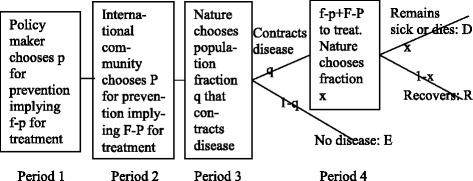
Four-period game for policy maker, international community, and nature

A four-period complete information game is considered as shown in Fig. 1. There are three players, i.e. the policy maker in a country equipped with funds *f*, the international community equipped with funds *F*, and nature. In period 1 the policy maker distributes an amount *p* of its funds to disease prevention, which implies that the remaining amount *f-p* gets distributed to disease treatment. In period 2, having observed the policy maker’s strategic choice in period 1, the international community distributes an amount *p* of its funds to disease prevention, which implies that the remaining amount *F-P* gets distributed to disease treatment. In period 3, affected by the policy maker’s choice p in period 1 and the international community’s choice P in period 2, nature chooses the population fraction q that contracts the disease. In practice, this means that each individual in the country observes the choices p and P and thereafter chooses a degree of risky behavior that affects the probability q that the individual contracts the disease.

If the disease contraction probability q is large, the policy maker and international community are more likely to distribute more funds f and F. The disease contraction probability q is assumed to decrease convexly in the prevention efforts p and P, and funds f and F, expressed probabilistically as

1 $$ \begin{array}{l} q= q\left( p,\chi P, f, F,{\delta}_1, nature\right),\kern0.5em \partial q/\partial p<0,\kern0.5em \partial q/\partial P<0,\kern0.5em {\partial}^2 q/\partial {p}^2>0,\kern0.5em {\partial}^2 q/\partial {P}^2>0,\kern0.5em \\ {}\partial q/\partial f<0,\kern0.5em \partial q/\partial F<0,\kern0.5em {\partial}^2 q/\partial {f}^2>0,\kern0.5em {\partial}^2 q/\partial {F}^2>0,\kern0.5em 0\le q\le 1\end{array} $$

where χ ≥ 0 expresses the efficiency of the international community relative to the country’s policy maker of converting funds into disease prevention or treatment, nature expresses that nature in the form of individuals make strategic choices based on the parameters and strategic choices in periods 1 and 2, and δ_1_ is a set of parameters specific for the country that impacts disease contraction. Examples of such parameters pertain to governmental systems, infrastructure, climate, culture, employment rate, income, education, etc. If χ > 1, the international community converts funds more effectively than the policy maker into prevention, and conversely if 0 ≤ χ < 1. The inequalities in equation (1) state that more funds p and *P* distributed to disease prevention, decreases the probability of disease contraction.

If the individual does not contract the disease, i.e. remains healthy, his/her utility is E. In contrast, if the individual contracts the disease and gets sick, the distributions f-p and F-P, implied by the strategic choices in period 1 and 2, come into play. The probability x that the average individual, having contracted the disease, remains sick or dies is assumed to decrease convexly in the disease treatment efforts f-p and F-P, and funds f and F, i.e.

2 $$ \begin{array}{l} x= x\left(\varepsilon \left( f- p\right),\varepsilon \chi \left( F- P\right),{\delta}_2, nature\right),\kern0.5em \partial x/\partial p>0,\kern0.5em \partial x/\partial P>0,\kern0.5em \\ {}{\partial}^2 x/\partial {p}^2<0,\kern0.5em {\partial}^2 x/\partial {P}^2<0,\kern0.5em \partial x/\partial f<0,\kern0.5em \partial x/\partial F<0,\kern0.5em {\partial}^2 x/\partial {f}^2>0,\kern0.5em {\partial}^2 x/\partial {F}^2>0,\kern0.5em 0\le x\le 1\end{array} $$

where ε ≥ 0 is the cost effectiveness of treatment relative to prevention, nature expresses that nature in the form of individuals make strategic choices based on the parameters and earlier strategic choices, and δ_2_ is a set of parameters specific for the country that impacts getting sick or dying. The set δ_2_ may be similar to the set δ_1_ in equation (1), but may differ to the extent that the mechanisms for getting sick or dying differ from the mechanisms of disease contraction. If ε > 1, funds distributed to treatment are utilized more effectively than funds distributed to prevention, and conversely if 0 ≤ ε < 1. The inequalities in equation (2) state that more funds f-p and F-P distributed to disease treatment decreases the probability of getting sick or dying. Each individual receives utility D if he/she remains sick or dies, and utility R if he/she recovers,^3^ where D < R < E.

Using Fig. 1, the average individual’s expected utility is

3 $$ v=\left(1- q\right) E+ q\left(\left(1- x\right) R+ xD\right)= E- q\left( E- R\right)- q x\left( R- D\right) $$

Modeling the policy maker’s funds f means accounting for the population size. Assuming n individuals in the country, and inserting equation (3), the policy maker’s expected utility is

4 $$ u= n v- a f= n\left(1- q\right) E+ n q\left(\left(1- x\right) R+ xD\right)- a f $$

where *a* is the unit cost of converting funding into utility for the n individuals. Analogously, the international community’s expected utility, inserting equation (3), is

5 $$ U= n v- A F= n\left(1- q\right) E+ n q\left(\left(1- x\right) R+ xD\right)- A F $$

where b is the unit cost of converting funding into utility for the n individuals.

Summing up, *f, F, χ, ε, δ*_1_, *δ*_2_, *a, b, E, R, D* are all exogenous parameters. Although funding f and F are exogenous in the model, these are allocated from national and international budgets which means that they in practice are chosen. As we discuss the model we will to some extent account for the possibility that f and F are chosen through mechanisms not specified in the model. In the model, the policy maker has one strategic choice variable, i.e. distribution p to prevention. Analogously, the international community has one strategic choice variable, i.e. distribution P to prevention. Nature in equations (1) and (2) means that nature in the form of individuals additionally impact the probability q of disease contraction and the probability *x* of remaining sick or dying given that the disease has been contracted.

## Methods and Results: Interpreting and analyzing the model

The game is solved with backward induction. For period 2, differentiating the international community’s expected utility U in (5) gives

6 $$ \frac{\partial U}{\partial P}=- n\underset{<0}{\frac{\partial q}{\partial P}}\left( E- R\right)- n\underset{<0}{\frac{\partial q}{\partial P}} x\left( R- D\right)- n q\underset{>0}{\frac{\partial x}{\partial P}}\left( R- D\right)=0 $$

and

7 $$ \frac{\partial^2 U}{\partial {P}^2}=- n\underset{>0}{\frac{\partial^2 q}{\partial {P}^2}}\left( E- R\right)- n\underset{>0}{\frac{\partial^2 q}{\partial {P}^2}} x\left( R- D\right)-2 n\underset{<0}{\frac{\partial q}{\partial P}}\underset{>0}{\frac{\partial x}{\partial P}}\left( R- D\right)- n q\underset{<0}{\frac{\partial^2 x}{\partial {P}^2}}\left( R- D\right)<0 $$

where the signs below the derivatives are from equations (1) and (2). Since ∂*q*/∂*P* < 0, the first two terms in equation (6) are positive. Since ∂*x*/∂*P* > 0, the third and last term in equation (6) is negative. Hence functional forms for q and x exist that enable an interior solution for P, i.e. 0 < P < F. This interior solution is such that the international community distributes a final amount P to prevention, and the remaining amount F-P to treatment. The first term in equation (6) expresses that increased distribution P to prevention causes decreased disease contraction probability q, which is multiplied with the utility difference E-R between no disease and recovery. The second term in equation (6) also expresses that increased P causes decreased q, but this derivative is multiplied with the probability x of remaining sick or dying, and multiplied with the utility difference R-D between recovery and being sick or dying. These first two terms considered in isolation show that the international community increases its expected utility U by increasing its distribution P to prevention. Without something to constrain the value of P, the international community would choose maximum P = *F*. The third term provides this constraint. That is, the third term in equation (6) expresses that increased distribution P to prevention causes increased probability x of remaining sick or dying. This derivative is multiplied with the disease contraction probability q and the utility difference R-D between recovery and being sick or dying. This third term thus provides a counterweight to the first two terms. The international community cannot be expected to choose maximum P = F for prevention which may give an unacceptably high probability x since no funds are distributed to treatment. This reasoning shows how the international community can be expected to strike a balance between prevention P and treatment F-P. Functional forms of q and x also exist that cause the second derivative in equation (7) to be negative, thus ensuring a solution P = P*(p). Inserting this into equation (4) and differentiating the policy maker’s expected utility u to determine the first period solution gives

8 $$ \frac{\partial u}{\partial p}=- n\underset{<0}{\frac{\partial q}{\partial p}}\left( E- R\right)- n\underset{<0}{\frac{\partial q}{\partial p}} x\left( R- D\right)- n q\underset{>0}{\frac{\partial x}{\partial p}}\left( R- D\right)=0 $$

where *q* = *q*(*p*, χP*(*p*), *δ*_1_, *nature*) and *x* = *x*(*ε*(*f* − *p*), *εχ*(*F* − P*(*p*)), *δ*_2_, *nature*) and

9 $$ \frac{\partial^2 u}{\partial {p}^2}=- n\underset{>0}{\frac{\partial^2 q}{\partial {p}^2}}\left( E- R\right)- n\underset{>0}{\frac{\partial^2 q}{\partial {p}^2}} x\left( R- D\right)-2 n\underset{<0}{\frac{\partial q}{\partial p}}\underset{>0}{\frac{\partial x}{\partial p}}\left( R- D\right)- n q\underset{<0}{\frac{\partial^2 x}{\partial {p}^2}}\left( R- D\right)<0 $$

The reasoning for the policy maker in period 1 to strike a balance between prevention p and treatment f-p is analogous to the reasoning for the international community in period 2 to strike a balance between prevention P and treatment F-P.

*Property 1: If the policy maker and the international community distribute unusually much funds p and P to prevention, the probability q of disease contraction is low, and most individuals receive the no disease utility E. Those few that contract the disease, have a high probability x of remaining sick or dying causing minimum utility D since limited funds f-p and F-P are distributed to treatment.*

Proof: Property 1 follows from inserting ∂*q*/∂*p* < 0 and ∂*q*/∂*P* < 0 into equation (3) which causes 1-q in the first term to be close to 1 and q in the second term to be close to 0.

Individuals differ with respect to risky behavior. Some may choose risky behavior regardless of prevention efforts p and P, and some of these will statistically contract the disease. This prevents q from ever reaching zero. Those that contract the disease are likely to remain sick or dying since limited funds are distributed to treatment. Conversely, some may choose safe behavior regardless of prevention efforts p and P. These are unlikely to contract the disease (except e.g. through receiving contaminated blood or being attacked with a contaminated needle). Aside from these two extremes, many individuals may vacillate between risky and safe behavior, to be impacted by p and P. The second part of Property 1 follows from inserting ∂*x*/∂*p* > 0 and ∂*x*/∂*P* > 0 into equation (3) which causes 1-x to be small and x to be large in the second term.

*Property 2: If the policy maker and the international community distribute limited funds p and P to prevention, and thus substantial funds f-p and F-P to treatment, the probability q of disease contraction is high, and few individuals receive the no disease utility E. The many that contract the disease, have a high probability 1-x of recovering causing utility R since substantial funds f-p and F-P are distributed to treatment.*

Proof: Property 2 is the opposite of Property 1, and follows from equation (3).

The balance to be struck between prevention and treatment also depends on the nature of the disease partly expressed with the utilities E,R,D, e.g. whether it is deadly, how long it lasts, whether individuals somehow recover themselves, and whether and through which channels it spreads to other individuals. For deadly diseases that spread easily, prevention seems more called for than for minor diseases which enable individuals to function close to normally.

*Property 3: If the cost effectiveness parameter ε of treatment relative to prevention is larger than 1, distributing more funds towards treatment is more appropriate.*

Proof: Follows from equation (2) and the discussion thereafter.

Diseases vary greatly in how they suggest prevention or treatment as the appropriate strategy. In the early history of HIV, when treatment options were few or absent expressed with ε close to zero, prevention was the preferred strategy since disease contraction in some cases was a death sentence. As treatment options become more readily available, and at an affordable price, so that ε increased, more funds to treatment seem appropriate to enable those having contracted the disease to recover and live their lives despite disease contraction.

*Property 4: If the country- specific parameter δ*_*1*_ *is such that the disease contraction probability q is high, more funds to prevention seems appropriate. Conversely, if the country specific parameter δ*_*2*_ *is such that the probability x of remaining sick or dying is high, more funds to treatment seems appropriate.*

Proof: Follows from equation (1) and the discussion thereafter for δ_1_, and from equation (2) and the discussion thereafter for δ_2_.

Property 4 suggests that different conditions in different countries may impact the balance policy makers and the international community strike between prevention and treatment.

*Property 5: If the efficiency χ of the international community relative to the country’s policy maker of converting funds into disease prevention or treatment is large, then the international community can be expected to contribute relatively more than the policy maker, especially if conditions require more funds.*

Proof: Follows from equation (1) and the discussion thereafter, and from equation (2).

Conditions vary greatly across countries regarding how funds are converted into disease prevention or treatment. Especially some third world countries, even if monetary funds are somehow available, may lack the competence, infrastructure, governmental institutions, equipment, transport capabilities, medicine, etc. to convert funds into prevention or treatment. In such instances, the international community, with more readily access to some of these capabilities expressed with large χ, may contribute more. However, the reverse may also occur. Local and cultural traditions, language, fear or non-acceptance of outsiders, etc. may hinder or block international community entry, thus expressing low χ, where the policy maker can be expected to contribute more.

*Property 6: The unit costs a and A, for the policy maker and international community respectively, of converting funding into utility for the n individuals impact their contributions.*

Proof: Follows from equation (4) for a and equation (5) for A.

Whereas *χ* in Property 5 expresses the funds conversion efficiency of the international community relative to the policy maker, the parameters a and A express how efficiently funds are converted into utility, and also provide scaling of the last terms af and AF in equations (4) and (5) relative to the first term nv.

*Property 7: As the recovery utility R and the utility D of remaining sick or dying decreases to be substantially below the no-disease utility E, decreasing the disease contraction probability q becomes more important and funding f and F increases.*

Proof: Follows from Fig. 1, the derivatives in equations (1) and (2), and the expected utilities in equations (4) and (5).

Property 7 illustrates the importance of accounting especially for the three utilities E, R and D at the end of period 4 in Fig. 1.

*Property 8: The policy maker or the international community may choose to free ride on their contributions p and P, f-p and F-P, and f and F, and even finance other needs, in the hope that the other player with contribute more to the funding needs of HIV.*

Proof: Differentiating equation (3) gives the positive derivatives

10 $$ \begin{array}{l}\frac{\partial v}{\partial f}=-\underset{<0}{\frac{\partial q}{\partial f}}\left( E- R\right)-\underset{<0}{\frac{\partial q}{\partial f}} x\left( R- D\right)- q\underset{<0}{\frac{\partial x}{\partial f}}\left( R- D\right)>0,\\ {}\frac{\partial v}{\partial F}=-\underset{<0}{\frac{\partial q}{\partial F}}\left( E- R\right)-\underset{<0}{\frac{\partial q}{\partial F}} x\left( R- D\right)- q\underset{<0}{\frac{\partial x}{\partial F}}\left( R- D\right)>0\end{array} $$

Differentiating the expected utilities u and U in equations (4) and (5) with respect to the other player’s funding, i.e. F and f respectively, and inserting equation (10), also gives positive derivatives, i.e.

11 $$ \frac{\partial u}{\partial F}=\frac{\partial }{\partial F}\left( nv- af\right)= n\frac{\partial v}{\partial F}>0,\kern1em \frac{\partial U}{\partial f}=\frac{\partial }{\partial f}\left( nv- AF\right)= n\frac{\partial v}{\partial f}>0 $$

Since both players benefit from increased funding by the other player, incentives for free riding exist.

Even if a policy maker has available funds, to prevention or treatment or both, he may choose to withhold these funds for strategic reasons in period 1, in order to induce the international community to contribute the required funds in period 2. Conversely, the international community may announce a small amount of F funds for a given country in the hope that the policy maker may live up to his commitment and provide the required funds.

## Methods and Results: Comparing the model with empirics

In this section we test aspects of the model against data from Africa, the region that is most affected by HIV and accounts for 78% of all people living with HIV. First we classify countries according to various characteristics. Second we run regressions in order to determine which characteristics matter in disease contraction.

## Introduction

In 2015 more than 36.7 million people lived with HIV/AIDS worldwide.^4^ Since its outbreak, 78 million people have become infected with HIV and 35 million people have died worldwide, of which 1.1 million died in 2015 alone. The situation is particularly acute in Africa where in 2015, 25.5 million were living with HIV. Eastern and southern Africa accounts for 46% of new HIV infections worldwide. In Africa 800.000 people died from AIDS-related illnesses in 2015. With more prevention measures, this number would be lower. But, with adequate treatment many can today live successfully with diseases such as HIV/AIDS.

With the advent of antiretroviral treatment (ART), HIV is no longer a death sentence but a chronic condition, with almost 13 million people in low and middle income countries now receiving ART. Treatment activities using ARTs are also preventative measures, as an individual who is well is not only alive and productive, but will also not transmit the disease. Prevention measures may also involve programs aimed at changing people’s behavior or changing cultural norms. While the trajectory of the HIV epidemic has begun to change with declining number of new infections and mortality levels, the cost trajectory has continued upwards, driven by lifetime treatment needs, longer living cohorts of individuals receiving treatment, expanded treatment guidelines, and rising prevention costs in HIV-affected countries which have expanding populations. The global resource need, which was US$ 3.8 billion in 2002, was US$19.1 billion in 2013. Recent estimates in Atun et al. [2] point to about US$22-24 billion being needed per annum, to fund HIV intervention programs.

The economic, social and health benefits of HIV investment have been well researched. Increased labor productivity, reduced orphan care costs, deferred treatment and end-of-life care have been estimated to produce substantial economic gains. Similarly, expanded services have been shown to benefit populations by strengthening health systems and releasing system capacity to treat other conditions. Adding to the mix are cross-sectorial benefits and social protection realized with prudent investment in HIV prevention and treatment. Those benefits nevertheless remain tenuous due to a chasm in financing. Prior modeling suggested that annual resource needs could reach US$35 billion by 2030.

While the resource needs are enormous, in many low-income countries, especially in Africa, domestic sources remain very low, with HIV co-financing dependent on external sources. There is also evidence of free riding by affected countries. However, the donor sources are now being constrained by the fiscal constraints from the global economic crisis. Inefficiencies in channeling and utilization of available funds, also adds to resource constraints. Clearly, there is a need to create mechanisms for commitment to funding by both affected countries and donors in order to avoid ‘free riding’ by both parties.

Domestic financing remains constrained in sub-Saharan Africa. In Nigeria, for example, domestic financing accounted for US$123 million in 2014 compared to US$451 million. From external financing for the same year. In Uganda external financing was US$446 million, nine times more than the US$53 million from domestic resources. In Malawi, external financing accounted for 98% of overall resources spent on HIV intervention.

## Country classification

Table 1 classifies countries according to the characteristics in (1) that determine the empirically estimated disease contraction probability q_e_, i.e. country characteristics δ_1_ (high δ_1_ expresses high disease contraction probability), the policy maker’s funds f, and the policy maker’s empirically estimated fraction p_e_ distributed to disease prevention. Furthermore, the rightmost three columns in Table 1 classifies countries according to the characteristics in (2) that determine the empirically estimated probability x_e_ that the average individual remains sick or dies, i.e. country characteristics δ_2_ (high δ_2_ expresses high probability that an individual remains sick or dies), the policy maker’s funds f, the policy maker’s empirically estimated fraction p distributed to disease prevention, and the international community’s empirically estimated funds provision F.

**Table 1:** Population size *n*, policy maker’s funds f, and the ratio δ_1_ impacting the disease contraction probability q for 43 countries. Policy makers’ empirically estimated strategic choice p_e_ that impact the empirically estimated disease contraction probabilities q_e_, where Int means Intermediate and Lo/I means Low/Int. Additionally, the ratio δ_2_ impacting the probability x that the average individual remains sick or dies, and the empirically estimated strategic choice F_e_ that impact the empirically estimated probabilities x_e_ that the average individual remains sick or dies^a^

Country	n^b^	f^c^	δ_1_^d^	p_e_^e^	q_e_	δ_2_^f^	F_e_^g^ _$ mill(%) 2009-2011_	x_e_^h^ (%)
Angola	21256000	High(18.8%)	Low	0.342’	Low	Low	20.45(0%)	0.061
Benin	9742000	Int(15.4%)	Low	0.134”	Low	Low	27.80(1%)	0.031
Botswana	2096000	High(35.2%)	High	0.096’	High	High	123.14(3%)	0.286
Burkina Faso	17323000	Int(11.5%)	Low	-	Low	Low	35.63(1%)	0.035
Burundi	9023000	Int(17.4%)	Low	0.203”	Low	Low	26.79(1%)	0.055
Cameroon	20930000	Int(18.3%)	Low	-	Low	Int	22.00(1%)	0.167
Chad	12948000	Int	Low	0.294”	Low	Int	15.12(0%)	0.108
Congo, Dem Rep	74618000	Int(13.2%)	Low	-	Low	Low	56.44(0%)	0.043
Cote d'Ivoire	23919000	Int(15.2%)	Low	-	Low	Int	80.54(2%)	0.130
Egypt	84605000	Int(15.8%)	Low	-	Low	-	-	-
Equatorial Guinea	1837000	Low(1.7%)	Int	-	Int	Low	1.06(1%)	0.054
Eritrea	4980000	-	Low	-	Low	Low	15.53(0%)	0.02
Ethiopia	86614000	Int(11.6%)	Low	-	Low	Low	367.59(8%)	0.054
Gabon	2204000	Int(10.3% + Oil)	Lo/I	0.167”	Low	Low	2.94(0%)	0.091
Gambia	1794000	Int(18.9%)	Low	-	Low	-	6.76(0%)	-
Ghana	26441000	High(20.8%)	Low	0.281’	Low	Low	51.80(0%)	0.045
Guinea	11861000	Low(8.2%)	Low	0.135”	Low	Low	8.49(0%)	0.042
Guinea-Bissau	1699000	Int(11.5%)	Lo/I	-	Lo/I	Int	6.24(0%)	0.118
Kenya	43291000	Int(18.4%)	Int	0.270’	Int	Int	425.86(10%)	0.132
Lesotho	1887000	High(15%)	High	-	High	High	52.70(1%)	0.795
Liberia	3881000	Int(13%)	Low	0.313’	Low	Low	12.90(0%)	0.052
Madagascar	21852000	Int(10.7%)	Low	0.515”	Low	Low	10.15(0%)	0.027
Malawi	15316000	High(20.7%)	High	0.113’	High	High	146.23(3%)	0.300
Mali	16678000	Int(15.3%)	Low	-	Low	Low	22.04(1%)	0.030
Mauritania	3461000	Int(12.9%)	Low	0.144”	Low	-	0.61(0%)	-
Mauritius	1273000	Int(19%)	Low	-	Low	-	1.58(0%)	-
Morocco	32950000	Int(13.4%)	Low	-	Low	Low	-	0.003
Mozambique	24491000	High(22.3%)	High	0.422”	High	High	240.32(5%)	0.314
Namibia	2170000	High(28.8%)	High	-	High	High	114.22(3%)	0.230
Niger	17493000	Int(11%)	Low	0.421”	Low	Low	11.52(0%)	0.017
Nigeria	177096000	Low(6.1%)	Low	-	Low	Int	401.22(9%)	0.136
Rwanda	10780000	Int(14.1%)	Low	-	Low	Low	187.99(4%)	0.056
São Tomé and Príncipe	194000	Int(17.4%)	Low	0.046”	Low	-	0.30(0%)	-
Senegal	13567000	Int(19.2%)	Low	0.383’	Low	Low	25.34(1%)	0.015
Sierra Leone	5823000	Lo/I(10.5%)	Low	-	Low	Low	17.83(0%)	0.052
South Africa	52982000	High(26.9%)	High	-	High	High	595.11(14%)	0.453
Swaziland	1077000	High(39.8%)	High	-	High	High	50.58(1%)	0.557
Tanzania	45950000	Int(12%)	Int	-	Int	Int	341.80(8%)	0.174
Togo	6675000	Int(15.5%)	Low	0.257”	Low	Int	14.20(0%)	0.105
Tunisia	10889000	Int(14.9%)	-	-	-	-	-	-
Uganda	35363000	Int(16.1%)	High	-	High	Int	284.60(7%)	0.178
Zambia	14129000	Int(16.1%)	High	-	High	High	255.15(6%)	0.212
Zimbabwe	13098000	High(49.3%)	High	0.152”	High	High	98.95(2%)	0.298

Notes: *f* is tax revenues as % of GDP. Low is 0-10%, Intermediate is 10.1-20%, and High is over 20%;’ and” denote 2011 and 2012 figures, respectively. - means data is not available. Figures for donor funding F are in US$ mill and percentage of total donor funding is in parenthesis. Figures for probability of remaining sick or dying x are in % probability; The ranges for δ_2_ are Low(less than 0.1%), Intermediate(between 0.0 and 2%) and High(above 2%)

^a^The data is sourced from World Health Organization(2014), UNAIDS (2014), The Global Fund for HIV/AIDs, Malaria and TB (2014), and the World Bank Statistical Data Base

^b^Data sourced from World Population Prospects, Economic and Social Affairs, United Nations, 2015

^c^Data sourced from the World Bank Data Base (2015)

^d^Constructed from data from the World Health Organization (WHO), 2015, and using a scale

^e^Data sourced from The Global Fund and UNAIDS, 2015

^f^Constructed from data from the World Health Organization (WHO), 2015, and using a scale. The ranges for δ_2_ are Low(less than 0.1%), Intermediate(between 0.0 and 2%) and High(above 2%)

^g^Data sourced from the Global Fund, 2015

^h^Estimated from data from the World health Organization (WHO), 2015

From Table 1 we see that countries with high resource mobilization, as measured by tax revenues to GDP ratio, f, are Angola, Botswana, Ghana, Lesotho, Mozambique, Namibia, South Africa, Swaziland and Zimbabwe. The bulk of the countries are in the intermediate stage of tax resource mobilization capacity. The countries with higher levels of tax mobilization capacity also have a higher HIV prevalence rate. These are also the countries with the highest levels of HIV contraction probability q_e._ This perhaps means that those countries with no extractive resource endowment, such as Swaziland and to some extent Lesotho, have no more room to raise taxes and require innovative finance solutions or indeed further external aid.

The probability of remaining sick or dying from HIV-related illness is highest (above 0.2%) in Botswana, Lesotho, South Africa, Swaziland, Namibia, Mozambique, Malawi, Zambia and Zimbabwe. This pattern corresponds to the probability of contracting the disease. The rest of the countries have moderate probability of death from the disease.

Looking at resource allocation between prevention and treatment, countries that are allocating more to prevention, i.e. high p_e_, are Angola, Chad, Ghana, Kenya, Liberia, Madagascar, Togo, Senegal, Mozambique, and Niger. Data on the split in resources between treatment and prevention of HIV is scarce.

Regarding external support from donors, Table 1 shows how they are contributing in each country. Contributions would be driven by many factors but one of them would be disease prevalence and capacity to manage the use of the resources. Countries with high and intermediate disease contracting probability receive a higher than average quantity of external aid.

## Testing the model using regression analysis

This section analyzes the model econometrically, starting with the disease contraction probability q in equation (1), expressed as incidence. We wish to determine which country characteristics matter, and we estimated a regression equation of HIV incidence (probability of contracting HIV), against country characteristics variables such as GDP per capita, literacy, Voice & accountability indicators, government effectiveness, inequality, and poverty levels. GDP per capita is expected to have a negative relationship with HIV incidence in the sense that poorer countries with low GDP per capita are likely to have weaker health delivery systems and therefore higher levels of HIV incidence. Literacy levels, particularly higher education, is also expected to have a negative relationship with HIV incidence due to the fact that education and campaigns on prevention measures is likely to be more effective in countries with higher literacy levels. Voice & accountability indicators are expected to have a negative relationship with prevalence due to the fact that a higher level of freedom of expression is a good medium for prevention campaigns. On accountability, the higher this indicator the more likely a country will improve service delivery. Higher general governance effectiveness is expected to have a negative relationship with incidence due to higher quality of service delivery to the population. Inequality levels are also expected to impact prevalence levels. The more unequal the society, the higher the prevalence level. Likewise, higher poverty levels are likely to be associated with a higher prevalence level, as the poor have lower access to medical care and are generally more vulnerable to disease. This is for 43 countries in Africa.^5^

Table 2 shows that only Inequality, International spending on HIV, Adult literacy, and Constant are significant. The most significant variable is international funding on HIV by financial donors. The Adult literacy rate is also quite significant and has a positive coefficient. However, this is the opposite of what is expected, but perhaps implies that middle-income countries, typically with a higher adult literacy levels, are exhibiting higher incidence levels due to other factors other than adult literacy. The countries just happen to be middle-income in classification. The inequality measure, i.e. the Gini coefficient, has a positive coefficient which is quite significant. This means that the more unequal the society, the higher the prevalence rate, as there is a large percentage of the population with poor access to health. Furthermore, inequality is quite high in some middle-income countries, which are showing higher levels of HIV incidence.

**Table 2:** Testing determinants of HIV incidence q in equation (1)

Variable	Coefficient	Std Error	t-Value	Significance level
Constant (Intercept)	1.012	0.558	1.814^b^	0.087
GDP per capita (current US$)	0.000	0.000	−0.603	0.555
Voice & Accountability	0.001	0.149	−0.009	0.993
Government Effectiveness	0.082	0.222	0.369	0.716
Adult Literacy Rate (%)	0.008	0.005	1.656	0.116
International Funding (US$1000)	0.022	0.006	3.656^a^	0.002
Poverty Head Count at US$1(%)	0.002	0.004	−0.598	0.558
GINI (Inequality)	0.023	0.014	1.698	0.108

^a^and ^b^mean significance at the 5% and 10% levels, respectively

The coefficient for GDP per capita is not significant, which means that the level of income for the country is no predictor for its level of HIV incidence. Some poor countries have low incidence rates, while some middle-income countries have some of the highest incidence rates in the world, such as Botswana, South Africa and Swaziland. Voice & accountability is not a driver of HIV incidence, as some of the countries with a relatively free environment for public expression exhibit high incidence rates, such as South Africa. Again, government effectiveness, which is a proxy for the quality if heath systems, is shown not to be a factor in driving the incidence rate. Equally, the level of poverty, as measured by headcount ratio, does not explain differentials in the incidence rate.

We continue with the probability x of remaining sick or dying in equation (2). Again we ran a regression to determine if indeed variables like the level of prevalence, population size, incidence rate, level of domestic funding and level of international funding were significant drivers of x. Table 3 shows that the incidence rate is the most significant determinant of likelihood of dying from HIV. Indeed, countries with a high incident rate exhibit a high probability of HIV death. The level of domestic funding also is significant. Here the negative sign shows that countries with low domestic funding seem to have a higher HIV death rate. This may imply that they need to commit more resources to HIV. Population size and the prevalence level do not seem to as highly correlated with HV death rates. Table 3 shows that a country’s HIV incidence rate is highly correlated with HIV deaths (t-Value = 7.773), as expected since HIV incidence is a necessary precursor to HIV deaths.

**Table 3:** Testing determinants of probability x of dying from HIV in equation (2)

Variable	Coefficient	Std Error	t-Value	Significance level
Constant	0.024	0.12	2.094^a^	0.048
Prevalence Level	−0.003	0.04	−0.871	0.393
Population Size (n)	3.629E-10	0.000	1.262	0.220
Incidence Rate	0.354	0.046	7.773^a^	0.000
International Funding	−5.001E-11	0.000	−0.644	0.526
Domestic Funding	−2.921E -10	0.000	−2.038^a^	0.053

R-Squared = 0.978, Adjusted R-Squared = 0.947; ^a^means significance at the 5% level

Next we consider international community funding F. From Table 4, the most significant driver of F is population size n. Table 3 shows that the higher the population size, the higher the aid flows. This may perhaps mean that the formula for allocating funds by donors relies too heavily on size of the country than the level of disease burden. If this is the case, this may need to be corrected.

**Table 4:** Determinants of international community funding F

Variable	Coefficient	Std Error	t-Value	Significance level
Constant	6822084.868	27527999.14	0.248	0.806
Prevalence(No)	6562456.103	10037639.62	0.654	0.519
Population Size(n)	2.643	0.503	5.244^a^	0.000
Incidence Rate(No)	21186583.86	115023421.114	−0.184	0.855
Domestic Funding	0.451	0.352	1.282	0.211

R-Squared = 0.757; Adjusted R-Squared = 0.572; ^a^ means significant at the 5% level

In terms of drivers of policy maker funding f, the prevalence rate, followed by the incidence rate, are major factors, as shown in Table 5. This makes sense, as one expects countries with high prevalence and incidence rate that feeds prevalence, to distribute more funding to HIV. The econometric results show that the drivers of the theoretic model do determine the outcomes.

**Table 5:** Determinants of policy maker funding f

Variable	Coefficient	Std Error	t-Value	Significance level
Constant	7408274.859	14835660.7	−0.499	0.622
Prevalence(No)	11851648.1	4955483.549	2.392^a^	0.024
Population Size(n)	0.252	0.388	−0.650	0.522
Incidence Rate(No)	97496309.5	59244152.819	−1.646	0.112
International Funding	0.132	0.103	1.282	0.211

R-Squared = 0.642; Adjusted R-Squared = 0.412; ^a^means significant at the 5% level

## Policy implications, suggestions and predictions

In this section we use the model and properties to generate policy implications and predictions.

*First*, when the policy maker has limited funding, the country has a large population, the disease contraction probability is large, and when the probability that an individual remains sick or dies is large, then the international community is more likely to contribute funds. This has largely been the case in countries such as Kenya or Mozambique or Uganda with low resources but high disease burden. This raises the issue of whether countries with small populations should be penalized and receive meager donor resources. One example is resource constrained Swaziland with a small population, and a high disease burden and incidence rate.

*Second*, if the international community announces willingness to provide funds, then the policy maker will free ride. This raises the issue of whether free riding by policy makers should be allowed, whether it should be regulated, etc. A few countries in Africa have abdicated on their duty of rescuing their citizens, and delegated HIV intervention to international donors. Country ownership of programs has been seen to help building domestic systems for service delivery. A further issue to consider is whether to broaden and deepen tax-bases and other revenue resources from affected countries. The relevance and strength of commitment mechanisms to regulate free riding practices by affected countries should be analyzed.

*Third*, if the policy maker has substantial available resources, e.g. Botswana with substantial revenues from natural resources, then an incentive exists for the international community to free ride and not provide funding. The wisdom of such free riding should be questioned. The international community has the capacity of imposing higher standards. The efficiency in converting funds into outcomes and utility is something that the international community has been emphasizing through pronouncements in the Paris Declaration and Aid-effectiveness pronouncements. Furthermore, even the countries with large resources are carrying the future debt from funding the liability of HIV. This debt needs to be financed, and even large domestic resources may not be enough.

*Fourth*, the probability of contracting the disease falls to its minimum when the policy maker distributes all his resources to disease prevention, and reaches a maximum when the policy maker distributes no resources to disease prevention. Obviously, when disease prevention works, it seems to make sense to distribute more resource towards it. However, the marginal cost of increasing coverage of prevention programs may rise as the policy maker adds more people to the programs. Especially including people in rural areas with poor road access may cause general service delivery costs to increase.

*Finally*, the probabilities of disease contraction, and remaining sick or dying given contraction, depend on the fractions of funds distributed to prevention versus treatment. More funding causes lower probabilities. One determining factor is the relative marginal costs of prevention versus treatment. One recommendation is to distribute funds to both activities by accounting for the relative marginal costs of provision, by equating the two marginal costs.

## Discussion and Conclusion

We have developed a four-period game between a policy maker and the international community on how to fund prevention and treatment of diseases, exemplified with HIV. We account for the behavior of individuals who engage in risky versus safe behavior which may or may not cause disease contraction. This approach is to our knowledge the first of its kind and extends earlier research where this phenomenon has been analyzed "one player at a time", instead of scrutinizing strategic interaction. The policy maker chooses in period 1 which fraction of his funds to distribute to disease prevention, and the remaining fraction is distributed to disease treatment. In period 2 the international community chooses an analogous distribution to prevention and treatment, or may choose not to provide funds. In period 3, nature determines who contracts the disease. Finally, in period 4, nature chooses, among those that have contracted the disease, who recovers and who remains sick or dies.

We find, first, that distributing substantial funds to prevention, by the policy maker or the international community, intuitively decreases the disease contraction probability, but those that contract the disease has a high probability of remaining sick or dying due to the limited funding to treatment. This illustrates how funding impacts how individuals behave in the face of a deadly disease. Second, and conversely, distributing substantial funds to disease treatment increases the disease contraction probability, but those contracting the disease recover with a higher probability. Third, if the cost effectiveness of treatment relative to prevention is higher, more emphasis on treatment is appropriate. Fourth, country specific conditions impact the appropriate relative channeling of funds into prevention versus treatment. Fifth, if the international community converts funds into disease prevention and treatment more efficiently than the policy maker, then relatively more funds from the international community may be expected in countries needing efficient funds conversion. Sixth, the policy maker’s and international community’s unit costs of converting funds into utility for the population impact their relative contributions. Seventh, more funding can be expected if the recovery utility and utility of remaining sick or dying are far below the no disease utility. Eighth, the policy maker and international community may free ride on each other’s contributions.

The disease contraction probability intuitively is minimal when the policy maker distributes all his funds to disease prevention, and maximal when no funds are distributed to disease prevention. Thus, for example, a large utility difference between no disease and recovery causes the policy maker to distribute a large fraction of his funds to disease contraction causing a low disease contraction probability. Furthermore, a large unit cost for the international community in converting funding into utility causes large disease contraction probability.

From outcomes of the model, the paper argues for the need to create commitment-mechanisms to ensure that free riding by both countries and the international community is avoided. This means replacing a non-cooperative game with a cooperative game. Examples of such commitment mechanisms are in the Global Fund for HIV/AIDS, TB, and Malaria. Such commitment mechanisms require countries to co-finance, which may be effective, as countries never fully co-finance. We furthermore argue for commitment to funding both prevention and treatment, by policy makers and the international community. Without commitment mechanisms the game may result e.g. in countries with limited resources suboptimally focusing only on prevention, or only on treatment. The model further illustrates the mechanisms for determining appropriate funding levels, acknowledging limited return on investment, and how more funding can reduce the probabilities of disease contraction and death from the disease.

We classify countries according to characteristics and strategic choices that determine the empirically estimated disease contraction probability. We also tested for the drivers for expressions for the fraction of resources distributed to disease prevention, the disease contraction probability, the probability that an individual remains sick or dies, and additional funding by the international community provides. We estimate the regression equations. The results largely confirm the various theoretical relationships. We discuss the policy implications of the outcomes (assertions) of the model. Future research should look more thoroughly into a "commitment technology" in the form of a global governance mechanism that forces policy makers and donors to both commit to funding prevention and treatment, and not to free ride.

## Footnotes

1 Examples are diseases which are infectious, airborne (pathogens may be viruses, bacteria, or fungi), non-communicable (e.g. heart disease, cancer), foodborne (consuming food with pathogenic bacteria, toxins, viruses, prions, parasites, etc.), or involve lifestyle (e.g. sedentary lifestyle, diets with refined carbohydrates, trans fats, alcohol, drugs, etc.).

2 We use the word international community to cover private and public financial donors, which also can come from within the given country from actors not associated with the country’s policy maker.

3 Recovery from a chronic disease such as HIV is impossible, but both prevention and treatment may ensure that the disease does not progress into AIDS causing death.

4
http://www.amfar.org/about-hiv-and-aids/facts-and-stats/statistics--worldwide/, retrieved January 18, 2017.

5 Angola, Benin, Botswana, Burkina Faso, Burundi, Cameroon, Chad, Congo, Democratic Rep., Cote d'Ivoire, Egypt, Equatorial Guinea, Eritrea, Ethiopia, Gabon, Gambia, Ghana, Guinea, Guinea-Bissau, Kenya, Lesotho, Liberia, Madagascar, Malawi, Mali, Mauritania, Mauritius, Morocco, Mozambique, Namibia, Niger, Nigeria, Rwanda, São Tomé and Príncipe, Senegal, Sierra Leone, South Africa, Swaziland, Tanzania, Togo, Tunisia, Uganda, Zambia, Zimbabwe.
